# Diverse Mechanisms and Circuitry for Global Regulation by the RNA-Binding Protein CsrA

**DOI:** 10.3389/fmicb.2020.601352

**Published:** 2020-10-27

**Authors:** Christine Pourciau, Ying-Jung Lai, Mark Gorelik, Paul Babitzke, Tony Romeo

**Affiliations:** ^1^ Department of Microbiology and Cell Science, Institute of Food and Agricultural Sciences, University of Florida, Gainesville, FL, United States; ^2^ Department of Biochemistry and Molecular Biology, Center for RNA Molecular Biology, The Pennsylvania State University, University Park, PA, United States

**Keywords:** RNA-binding proteins, sRNAs, posttranscriptional regulation, regulatory circuitry, translation control

## Abstract

The carbon storage regulator (Csr) or repressor of stationary phase metabolites (Rsm) system of *Gammaproteobacteria* is among the most complex and best-studied posttranscriptional regulatory systems. Based on a small RNA-binding protein, CsrA and homologs, it controls metabolism, physiology, and bacterial lifestyle decisions by regulating gene expression on a vast scale. Binding of CsrA to sequences containing conserved GGA motifs in mRNAs can regulate translation, RNA stability, riboswitch function, and transcript elongation. CsrA governs the expression of dozens of transcription factors and other regulators, further expanding its influence on cellular physiology, and these factors can participate in feedback to the Csr system. Expression of *csrA* itself is subject to autoregulation *via* translational inhibition and indirect transcriptional activation. CsrA activity is controlled by small noncoding RNAs (sRNAs), CsrB and CsrC in *Escherichia coli*, which contain multiple high affinity CsrA binding sites that compete with those of mRNA targets. Transcription of CsrB/C is induced by certain nutrient limitations, cellular stresses, and metabolites, while these RNAs are targeted for degradation by the presence of a preferred carbon source. Consistent with these findings, CsrA tends to activate pathways and processes that are associated with robust growth and repress stationary phase metabolism and stress responses. Regulatory loops between Csr components affect the signaling dynamics of the Csr system. Recently, systems-based approaches have greatly expanded our understanding of the roles played by CsrA, while reinforcing the notion that much remains to be learned about the Csr system.

## Introduction and History

The *csrA* gene was discovered in studies aimed at finding regulators of gene expression in the stationary phase of bacterial growth. These studies used random transposon mutagenesis to identify trans-acting factors affecting glycogen biosynthesis and expression of a *glgC*’-‘*lacZ* translational reporter (Romeo and Gong, 1993; Romeo et al., 1993). At that time, transcript initiation was understood to be a focal point of genetic regulation, while translational regulation and posttranscriptional regulation were limited to a few examples, such as feedback inhibition of ribosomal protein synthesis (discussed in Babitzke et al., 2009, 2019; Vakulskas et al., 2015). Nevertheless, early observations on the regulation of *glgC* by CsrA began to associate this protein with posttranscriptional control, which involves CsrA-mediated repression associated with binding to the *glgC* untranslated mRNA leader, inhibition of ribosome loading, and mRNA destabilization (Liu et al., 1995, 1997; Liu and Romeo, 1997; Baker et al., 2002). The global regulatory roles of CsrA and its orthologs, e.g., RsmA, that began to emerge soon after its discovery included widespread effects on carbon metabolism, virulence of plant and animal pathogens, motility, and surface properties that mediate biofilm formation (Romeo et al., 1993; Sabnis et al., 1995; Mukherjee et al., 1996; Yang et al., 1996; Altier et al., 2000; Wei et al., 2001; Jackson et al., 2002). These and other roles of CsrA rely on the RNA-binding properties of this protein. The varied regulatory mechanisms mediated by CsrA depend on the positioning of its binding sites in target RNAs relative to other cis-acting elements and the ability of CsrA binding to alter RNA structure.

Purification of CsrA protein revealed its tight association with RNA, a large fraction of which consisted of a noncoding sRNA designated CsrB (Liu et al., 1997). The functions of this sRNA and a second similar one, CsrC (Weilbacher et al., 2003), defined a new paradigm of genetic regulation, in which a noncoding RNA molecule employs multiple high-affinity binding sites to sequester an mRNA-binding regulatory protein away from its target mRNAs. Predictably, genes and conditions that influence the biosynthesis and turnover of CsrB/C sRNAs affect the expression of genes in the CsrA regulon, although feedback in the system can sometimes obscure findings from single gene disruptions. The regulatory factors that control carbon storage regulator (Csr) sRNAs inform our understanding of how metabolic status and physiological conditions can impact translation and posttranscriptional processes to guide bacterial lifestyle decisions. This review focuses on regulatory mechanisms and circuitry involving CsrA and the Csr system of *Escherichia coli*, with occasional reference to homologous systems in other species.

## CsrA Structure and Sequence-Specific RNA Binding

CsrA is a small, highly conserved protein that is 61 amino acids in length in *E. coli*. Although early observations suggested a role for CsrA in posttranscriptional gene regulation, its amino acid sequence was originally found to be unrelated to known proteins and its structure and regulatory mechanism were unclear (Romeo et al., 1993; Liu et al., 1995). A major advancement in understanding its structure occurred when the 3D structures of three CsrA/RsmA family proteins were independently solved (Gutiérrez et al., 2005; Rife et al., 2005; Heeb et al., 2006). These structures revealed a novel RNA-binding topology and confirmed previous studies, indicating that *E. coli* CsrA functions as a homodimer (Dubey et al., 2003). In the active dimer, the CsrA monomer polypeptides exhibit a β-β-β-β-β-α secondary structure containing five consecutive antiparallel β-strands (β1–β5) and an α-helix, followed by a flexible C-terminus. The β-strands of each monomer interdigitate with those of the other monomer so that the dimer contains two interlocking five-stranded antiparallel β-sheets. The three central strands (β2–β4) of each β-sheet originate from one subunit while the two peripheral strands (β1, β5) come from the other subunit. The interlocking β-strands form a hydrophobic core from which the two α-helices extend.

**Figure 1:**
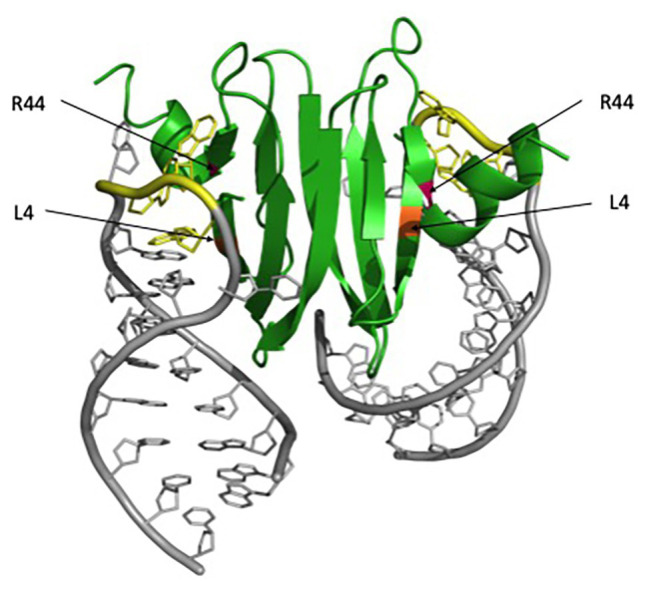
RsmA/CsrA binding to *hcnA* mRNA. The RsmA/CsrA protein is shown in green, critical RNA-binding residues are marked with asterisks, L4 is highlighted in orange, and R44 in magenta. The *hcnA* mRNA is shown in gray, with GGA motifs associated with binding highlighted in yellow. The figure was generated in PyMol using the Protein Data Bank (PDB) file 2JPP.

Alanine scanning mutagenesis of *E. coli* CsrA revealed two identical surfaces, located on opposite sides of the protein, which are essential for RNA binding and regulation (Mercante et al., 2006). These two surfaces are comprised of the parallel β1 and β5 strands of opposing monomers, which form two positively charged subdomains and can allow the binding of two RNA molecules simultaneously. (Schubert et al., 2007; Mercante et al., 2009; Figure 1). Highly conserved residues L4 in β1 and R44 in β5 are particularly critical for CsrA-RNA interaction. R44A substitution in *E. coli* CsrA resulted in defective RNA binding and eliminated regulation of target genes (Mercante et al., 2006). R44 is also important for RNA binding by the *Yersinia enterocolitica* CsrA (Heeb et al., 2006) and *Pseudomonas fluorescens* RsmE regulation (Schubert et al., 2007). L4A substitution resulted in a partial loss of regulation by RsmE in *P. fluorescens* as well (Schubert et al., 2007). These structural and functional studies established CsrA and its homologs as a novel class of RNA-binding protein, containing a new RNA-binding fold that mediates the posttranscriptional regulation of gene expression.

The first insights into the features of RNA that facilitate CsrA binding came with the identification of the noncoding sRNA CsrB, which interacts with CsrA and antagonizes its regulatory activity. The stoichiometry of the CsrA:CsrB complex indicated that each CsrB molecule binds ~18 CsrA subunits, forming a large globular ribonucleoprotein complex (Liu et al., 1997). The CsrB sRNA contained about the same number of repeated CAGGA(U/A/C)G sequence elements, suggestive of their function as targets for CsrA binding. These repeated sequence motifs were located within the loops of predicted hairpin structures or other single-stranded regions (Romeo, 1998). These early studies strongly suggested that a single-stranded GGA motif located within the loop of a hairpin structure serves as an important part of the CsrA binding site.

A more detailed exploration of the role of RNA sequence and secondary structure in CsrA binding was carried out using systematic evolution of ligands by exponential enrichment (SELEX). SELEX allowed the identification of high-affinity CsrA:RNA interactions from a large combinatorial RNA library. The SELEX-derived consensus sequence for a CsrA binding site was deduced to be RUACARGGAUGU. The ACA and GGA motifs were 100% conserved and the GU sequence was present in all but one ligand. Of the 55 RNAs identified, a majority (51) contained a GGA motif in the loop of a hairpin that was the most stable predicted structure, similar to the repeated elements found in CsrB. Most of the SELEX hairpins contained four or fewer contiguous base pairs, suggesting that very stable secondary structure might be disadvantageous for CsrA binding. Additionally, a majority (44) of the hairpin loops consisted of six nucleotides, with the upstream AC and downstream GU residues base-pairing together (Dubey et al., 2005). The studies also established that CsrA binding affinity is influences by both RNA primary sequence information and its structural context.

Solution nuclear magnetic resonance (NMR) of the CsrA homolog RsmE confirmed that differences in RNA sequence and structure can alter RsmE binding affinity and revealed that interaction of RNA with this protein introduces RNA curvature (Duss et al., 2014a,b). NMR structures of RsmE bound to high-affinity RNA targets revealed contacts involving a conserved hairpin structure containing an ANGGAN hexanucleotide loop. Hydrogen bond formation between the conserved A and GGA RNA bases and the RsmE backbone carbonyl and amino groups also occurs (Schubert et al., 2007). The N nucleotides of the sequence are variable, yet they affect binding affinity. The presence of additional nucleotides within the loop can decrease RsmE binding affinity, as can alterations in secondary structure, such as the absence of a hairpin structure or the involvement of the GGA motif in base pairing (Duss et al., 2014a).

Because each CsrA dimer contains two positively charged RNA-binding surfaces, and because many RNA targets of CsrA contain two or more binding sites, it was proposed that a CsrA protein might be able to interact simultaneously with two sites in a single transcript (Mercante et al., 2009). To explore this hypothesis, CsrA dimers with mutations (R44A) in one or both polypeptides, which alter one or both RNA-binding surfaces of the protein, were tested for interaction with RNAs containing two or more CsrA binding sites. Gel shift assays and other studies confirmed that CsrA is capable of “bridging” two sites on a single RNA that are separated by ≥10 to ≤63 nucleotides (nt), with an optimal intersite distance of >18 nt. Additionally, *in vitro* coupled transcription-translation reactions demonstrated that full repression of *glgC* requires a CsrA dimer to contain both of the functional RNA-binding pockets. Disruption of CsrA dual-site binding by RNA sequence alterations also interfered with regulation (Mercante et al., 2009). However, the requirement of two CsrA binding sites for full regulatory capability is apparently not essential, as one known CsrA target mRNA contains a single CsrA binding site (Baker et al., 2007). Structural studies using NMR and electron paramagnetic resonance spectroscopy (EPR) confirmed dual-site binding by the CsrA homolog RsmE. Furthermore, this protein bound to the individual sites within a multi-site sRNA target in a sequential order and with cooperativity (Duss et al., 2014b).

## CsrA Regulatory Mechanisms

### Repression by CsrA and Its Orthologs

**Figure 2:**
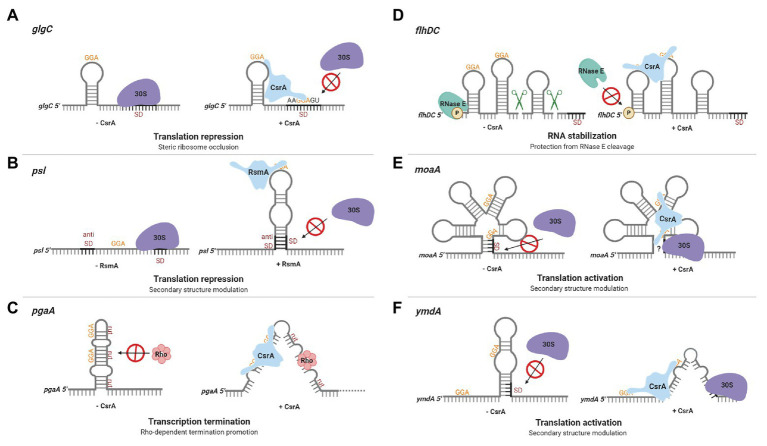
Regulation of gene expression by CsrA-family proteins. **(A)** CsrA repression of *glgC* translation in *Escherichia coli*. A CsrA homodimer binds to two sites in the *glgC* leader, a high-affinity site in a hairpin loop and a lower-affinity region overlapping the Shine-Dalgarno (SD) sequence, competing with ribosome (30S) binding and repressing translation. **(B)** Repression of *psl* translation in *Pseudomonas aeruginosa* by RsmA. The *psl* leader contains an unstable RNA hairpin formed by the SD and anti-SD regions. RsmA binding stabilizes the hairpin, prevents ribosome binding, and represses translation. **(C)** CsrA promotes Rho-dependent *pgaA* transcription termination in *E. coli*. In the absence of CsrA, the *pgaA* transcript forms a hairpin that sequesters Rho utilization (*rut*) sites. CsrA binding to two sites prevents this hairpin from forming, exposes the *rut* sites, and facilitates termination. **(D)** CsrA stabilization of the *flhDC* transcript in *E. coli*. CsrA binds to two sites located near the 5' end of the *flhDC* transcript, thus protecting this mRNA against RNase E cleavage and turnover. **(E)** CsrA activates *moaA* translation in *E. coli*. CsrA binds to two sites within an apparent Moco-responsive riboswitch aptamer in the *moaA* transcript. Binding at this location is proposed to expose the SD for ribosome binding, permitting translation. **(F)** CsrA activates *ymdA* translation in *E. coli*. In the absence of CsrA, *ymdA* mRNA forms a hairpin that sequesters the SD sequence. CsrA binding to a site within the hairpin and another site just upstream destabilizes the hairpin and permits ribosome access. These regulatory mechanisms are discussed in the text. This figure was created with BioRender.com.

The identification of CsrA as a regulator of *glgC* expression in *E. coli* led to the first insights regarding CsrA regulatory mechanisms. *glgC* mRNA is destabilized by CsrA, and deletion studies of a *glgC*’-‘*lacZ* translational fusion revealed that CsrA-mediated regulation was dependent on the region surrounding the *glgC* initiation codon and did not require a *glgC* promoter (Liu et al., 1995). *In vitro* experiments with purified recombinant protein confirmed that CsrA binds specifically to *glgC* mRNA and inhibits expression posttranscriptionally (Liu and Romeo, 1997). Further exploration demonstrated that CsrA binds four sites in the untranslated *glgC* leader (Baker et al., 2002). The downstream binding site overlaps the Shine-Dalgarno (SD) sequence, while a second binding site lies within the single stranded loop of an RNA hairpin that is located just upstream of the SD sequence. High-affinity binding to the *glgC* hairpin tethers the CsrA protein to this mRNA and permits the CsrA dimer to bridge to the SD sequence, where it prevents translation (Mercante et al., 2009; Figure 2A). Because translation and mRNA stability are often subject to coordinate regulation, translational inhibition likely contributes to CsrA-mediated instability of the *glgC* transcript (Liu et al., 1995; Vytvytska et al., 2000).

Subsequent investigation of other CsrA targets revealed that translation inhibition by ribosome exclusion is a common mechanism of CsrA-mediated gene regulation. *cstA*, which encodes a protein involved in peptide transport during carbon starvation in *E. coli*, was identified as a putative CsrA target based on sequence identity to the *glgC* leader. Much like *glgC*, the *cstA* leader contains up to four CsrA binding sites, with one overlapping the SD sequence. Toeprinting analysis of CsrA and 30S ribosomal subunit association with *cstA* RNA demonstrated that bound CsrA sterically inhibits ribosome binding and represses the expression of this gene (Dubey et al., 2003). The same mechanism has been reported in gram-positive bacteria. In *Bacillus subtilis*, CsrA represses translation of the gene encoding the flagellin protein (*hag*) by binding to two sites in the *hag* leader mRNA. One of these sites overlaps the SD sequence and CsrA bound at this location blocks ribosome binding (Yakhnin et al., 2007). Similar mechanisms of translational repression occur in many other mRNA targets, with CsrA and its homologs binding to regions overlapping the SD sequence, start codon, and/or initially translated region (Wang et al., 2005; Martinez et al., 2011; Yakhnin et al., 2011a; Pannuri et al., 2012; Abbott et al., 2015; Park et al., 2015; Pourciau et al., 2019). A unique example of CsrA-dependent ribosome exclusion is the repression of *sdiA*, which encodes the *N*-acyl-L-homoserine lactone receptor in *E. coli* (Dyszel et al., 2010). Unlike other known CsrA targets, the two CsrA binding sites in the *sdiA* transcript are located entirely within the coding region, although the upstream CsrA binding site overlaps the ribosome-binding segment of this transcript (Yakhnin et al., 2011a).

In *Pseudomonas aeruginosa*, the CsrA homolog RsmA represses translation of the *pslA* mRNA by sequestering the SD sequence *via* an alternative mechanism (Irie et al., 2010). The *psl* locus is responsible for the production of a biofilm supporting exopolysaccharide and the corresponding transcript has an extensive untranslated leader. RsmA binds to a single stranded site within the loop of a hairpin in the *psl* leader. Base pairing in this imperfect hairpin occurs between the SD and anti-SD sequences and is unstable in the absence of RsmA. RsmA binding to the transcript stabilizes the hairpin, which prevents ribosome binding and represses translation (Irie et al., 2010; Figure 2B). A more recently discovered mechanism of CsrA-mediated repression involves translational coupling, wherein the translation of a gene depends on the translation of a closely located upstream gene (Babitzke et al., 2009). CsrA regulates expression of *iraD*, which encodes an anti-adapter protein that inhibits RssB-mediated RpoS proteolysis in *E. coli*. The *iraD* leader contains four CsrA binding sites, but CsrA binding does not directly inhibit ribosome access to the *iraD* translation initation region. Instead, CsrA represses translation of a short open reading frame upstream from and translationally coupled to *iraD*, thereby repressing *iraD* expression (Park et al., 2017).

There are six CsrA binding sites within the *E. coli pgaA* noncoding leader, which are involved in multiple regulatory mechanisms (Wang et al., 2005). The *pgaABCD* operon encodes proteins responsible for synthesis and secretion of a biofilm polysaccharide adhesin consisting of a partially *N*-deacetylated polymer of β-1,6-*N*-acetyl-D-glucosamine (Wang et al., 2004, 2016; Itoh et al., 2008). CsrA interacts with the two sites overlapping the SD sequence and start codon, which inhibits ribosome binding and initiation of translation. Mutation of these binding sites relieved ~60% of CsrA-dependent repression (Wang et al., 2005). Studies of the remaining sites revealed a mechanism whereby CsrA binding promotes Rho-dependent transcription termination. In the absence of CsrA, the *pgaA* leader forms a hairpin that sequesters Rho utilization (*rut*) sites. CsrA binding to two sites in this region prevents hairpin formation, exposes the *rut* sites, and facilitates Rho-dependent termination (Figueroa-Bossi et al., 2014; Figure 2C). The exceedingly sophisticated direct regulation of *pgaABCD* expression by CsrA is striking, as it affects transcript elongation, translation, and transcript stability (Wang et al., 2005). However, given that many other CsrA RNA targets apparently possess multiple CsrA binding sites, CsrA may regulate expression of other genes using similarly complex mechanisms.

### Activation of Gene Expression by CsrA and Orthologs

Despite the prevalence of CsrA-mediated repression mechanisms, CsrA was recognized early as an activator of glycolytic gene expression *via* undetermined mechanisms (Sabnis et al., 1995). In addition, CsrA was found to activate the expression of *flhDC*, the master operon responsible for eliciting the motility and chemotaxis gene expression cascade in *E. coli* (Wei et al., 2001). The *flhDC* operon encodes the subunits of a DNA-binding protein (FlhD_4_C_2_) that recognizes class II flagellar promoters. In the absence of CsrA, the 5' monophosphorylated end of the *flhDC* transcript is accessible to RNase E, which binds to and cleaves this mRNA, facilitating its turnover. CsrA binding to two sites located near the 5' end of the *flhDC* transcript protects it against RNase E attack and stabilizes this mRNA (Yakhnin et al., 2013; Figure 2D).

CsrA also activates mRNA translation. In *Pseudomonas aeruginosa*, the CsrA homolog RsmA differentially regulates the expression of two genes involved in the biosynthesis of phenazine, *phz1* and *phz2*. RsmA represses the translation of *phz1* and activates the translation of *phz2*. RsmA-mediated activation of *phz2* seems to occur by a mechanism, which is opposite to that of *psl* repression in some respects. RsmA binding to the *phz2* leader is hypothesized to prevent the formation of secondary structure that sequesters the SD sequence, thus activating *phz2* translation (Ren et al., 2014).

CsrA also activates translation of *moaA* in *E. coli* by altering RNA structure. The *moaABCDE* operon encodes proteins required for molybdenum cofactor (Moco) biosynthesis. Translation of *moaA* is apparently inhibited *via* a Moco-responsive riboswitch aptamer, which is thought to sequester the SD sequence (Regulski et al., 2008). CsrA binding to two sites within the aptamer seemingly alters the structure to expose the SD to ribosome binding (Patterson-Fortin et al., 2013; Figure 2E). To our knowledge, these studies of *moaA* regulation by CsrA brought to light the first example of dual posttranscriptional regulation mediated by the binding of a cofactor (Moco) and an RNA-binding protein to the aptamer domain of a riboswitch.

The first detailed molecular mechanism for CsrA-mediated translational activation was recently established in a study exploring the expression of the uncharacterized *E. coli* protein YmdA. The *ymdA* gene was identified as the most strongly activated direct mRNA target of CsrA in the *E. coli* genome in an integrated transcriptomics study (Potts et al., 2017) and may play a role in biofilm formation (Kim and Kim, 2017). Reporter assays and mRNA half-life studies confirmed that CsrA posttranscriptionally activates *ymdA* expression and stabilizes the *ymdA* transcript (Renda et al., 2020). In the absence of CsrA, *ymdA* mRNA forms a hairpin that sequesters the SD sequence, inhibiting ribosome binding. CsrA-*ymdA* RNA footprint experiments revealed two CsrA binding sites in the *ymdA* leader, one located within the *ymdA* hairpin and the other just upstream of it. Disruption of CsrA binding by a mutation at either position eliminated CsrA-dependent activation *in vivo*. Toeprinting analysis of CsrA and 30S ribosomal subunit association with the *ymdA* transcript further revealed that CsrA binding destabilizes the SD-sequestering hairpin, permitting ribosome access and translation (Figure 2F).

## Regulation By CsrA Antagonists

sRNAs participate in multiple regulatory networks, allowing bacteria to rapidly alter gene expression in response to environmental cues in order to make lifestyle decisions (Mehta et al., 2008; Gottesman and Storz, 2011; Wagner and Romby, 2015; Nitzan et al., 2017). While most of the well-characterized bacterial sRNAs function by base pairing with their mRNA targets, the discovery of CsrB and CsrC RNAs of *E. coli* introduced sRNAs that act by binding to and inhibiting the activities of an RNA-binding regulatory protein (Liu et al., 1997, 1998; Romeo, 1998; Heeb et al., 2002; Valverde et al., 2003; Weilbacher et al., 2003; Kay et al., 2005; Babitzke and Romeo, 2007). CsrB from *E. coli* contains 22 GGA sequences, most of which serve as CsrA binding sites (Liu et al., 1997; Vakulskas et al., 2015, 2016). Another *E. coli* sRNA, CsrC, is structurally and functionally related to CsrB (Weilbacher et al., 2003). Phylogenetic analyses and other studies suggest that sRNAs in the CsrB family are widespread in *Gammaproteobacteria* and control genes responsible for a myriad of metabolic pathways and physiological functions (Zere et al., 2015; Potts et al., 2018), as well as complex traits such as biofilm formation (Jackson et al., 2002; Parker et al., 2017), host-microbe interactions, and pathogenesis (reviewed in Vakulskas et al., 2015).

Synthesis of the *E. coli* CsrB and CsrC sRNAs depends on the two-component signal transduction system (TCS) BarA-UvrY (Suzuki et al., 2002). Orthologs of BarA-UvrY are known by a variety of names and activate the synthesis of CsrA-inhibitory sRNAs in many *Gammaproteobacteria* (Pernestig et al., 2001; Vakulskas et al., 2015; Zere et al., 2015). Metabolic end products, namely short chain carboxylate compounds such as formate and acetate (R-COOH), stimulate the kinase activity of BarA, which phosphorylates the DNA binding response regulator, UvrY. BarA also possesses phosphatase activity, as deduced by genetics experiments (Camacho et al., 2015). The phosphorylated form of UvrY binds to DNA and activates transcription from the *csrB* and *csrC* promoters (Pernestig et al., 2001; Chavez et al., 2010; Camacho et al., 2015; Zere et al., 2015). Other activators of CsrB/C synthesis include the stringent response factors, ppGpp and DksA, and two DEAD-box RNA helicases, DeaD (CsdA) and SrmB (Edwards et al., 2011; Vakulskas et al., 2014; Zere et al., 2015). DeaD helicase functions by overcoming inhibitory basepairing within *uvrY* mRNA to activate translation, while SrmB stimulates *csrB/C* expression by unknown mechanisms.

**Figure 3:**
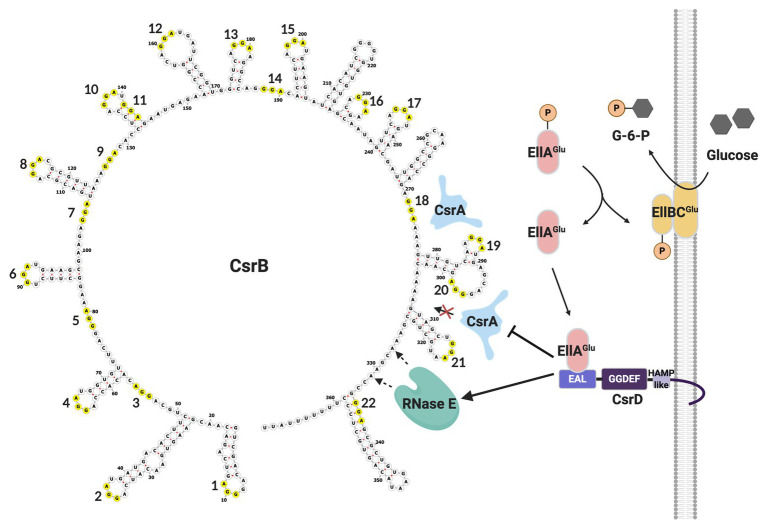
CsrB sRNA secondary structure and decay pathway. GGA motifs are numbered and circled in yellow. As glucose is transported into the cell, EllA^Glu^ protein becomes dephosphorylated and able to bind to the EAL domain of CsrD, potentiating CsrB decay. In the absence of CsrD-EIIA^Glc^, CsrA binding to CsrB protects it from RNase E cleavage and turnover. This figure was created with BioRender.com.

Turnover of CsrB/C sRNAs in *E. coli* is tightly regulated. In addition to the housekeeping endonuclease RNase E and the 3'-5' exonuclease polynucleotide phosphorylase (PNPase), it requires a non-nucleolytic EAL-GGDEF domain-containing protein, CsrD or MshH (Suzuki et al., 2006; Leng et al., 2016; Vijayakumar et al., 2018). Unlike classical EAL-GGDEF domain proteins, CsrD neither synthesizes nor degrades the signaling molecule (3'-5')-cyclic-diguanylate (c-di-GMP). Instead, the availability of a preferred carbon source, such as glucose, results in dephosphorylation of EIIA^Glc^ of the PTS pathway, which can then bind to the EAL domain of CsrD and trigger CsrB/C decay (Figure 3). This binding interaction is part of a complex membrane-localized mechanism that is required for turnover of these sRNAs in the presence of CsrA (Suzuki et al., 2006; Leng et al. 2016; Vakulskas et al., 2016; Hadjeras et al., 2019). CsrA binding to CsrB RNA blocks cleavage by RNase E at an unstructured site just upstream of the intrinsic terminator, which is necessary for initiation of turnover. The CsrD-EIIA^Glc^ interaction overcomes the inhibitory activity of CsrA (Leng et al., 2016; Vakulskas et al., 2016). While it seems that most or perhaps all *Gammaproteobacteria* express sRNAs that antagonize CsrA activity, CsrD orthologs appear to be restricted to the *Enterobacteriaceae*, *Shewanellaceae*, and *Vibrionaceae*. Interestingly, in contrast to short half-lives of CsrB/C in *E. coli*, Csr family sRNAs in species that lack a CsrD homolog are relatively stable, with half-lives reported from ∼20 min to >60 min (Valverde et al. 2004; Sonnleitner et al., 2006; Vakulskas et al., 2016). Moreover, the stability of the latter sRNAs is decreased in the absence of a CsrA homolog, while disruption of *csrA* has little or no effect on CsrB/C decay in *csrD* wild type (WT) strains of *E. coli* (Gudapaty et al., 2001). Thus, the evolution of CsrD was proposed to have provided a mechanism that allows the turnover of Csr sRNAs in *Enterobacteriaceae* and close relatives to respond to nutritional status. Viewed comprehensively, CsrB/C decay in *E. coli* is enhanced when preferred carbon sources are available, while their synthesis is activated by end products of metabolism (Chavez et al., 2010; Leng et al., 2016; Vakulskas et al., 2016).

CsrB, CsrC, as well as two basepairing sRNAs, McaS and GadY, have been reported to activate biofilm formation when they are overexpressed. This effect on biofilm was proposed to occur because these sRNAs bind to and antagonize CsrA activity, thus increasing *pgaA* expression (Wang et al., 2005; Itoh et al., 2008; Pannuri et al., 2012; Jørgensen et al., 2013; Parker et al., 2017). The basepairing sRNAs contain at least two GGA motifs and bind to CsrA with high affinity *in vitro* (Jørgensen et al., 2013; Potts et al., 2017). UV-crosslinking immunoprecipitation and sequencing (CLIP-seq) revealed that CsrA also interacts directly with other sRNAs *in vivo* in both *Salmonella* and *E. coli* (Holmqvist et al., 2016; Potts et al., 2017). The physiological functions associated with these interactions remain to be elucidated. Interestingly, CsrA was recently reported to interact with and promote pairing of the sRNA SR1 with the mRNA *ahrC* in *B. subtilis* (Müller et al., 2019). In addition, the CsrA homolog RsmA in *P. aeruginosa* has been shown to sometimes bind to nascent mRNAs in conjunction with the sRNA-mRNA chaperone protein Hfq (Gebhardt et al., 2020). These findings warrant additional studies and hint that CsrA may play important roles in sRNA-mRNA or other RNA-RNA interactions.

**Figure 4:**
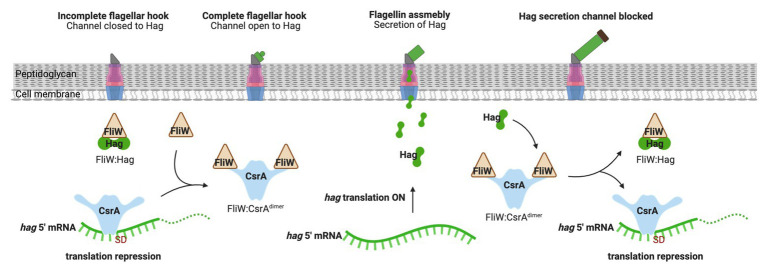
Regulation of flagellum biosynthesis in *Bacillus subtilis* by a partner-switching mechanism involving CsrA, Hag, and FliW. Prior to flagellar hook assembly, FliW is bound in a complex with cytoplasmic Hag (flagellin) protein and free CsrA represses *hag* translation by occluding ribosome access. Once assembly of the flagellar hook structure is completed, Hag is actively secreted to assemble the flagellum filament and FliW is released. Free FliW binds to CsrA, derepessing *hag* translation to allow flagellin synthesis. Upon capping of the channel, intracellular levels of Hag increase. Hag then binds to FliW, which releases CsrA to inhibit *hag* translation. This figure was created with BioRender.com.

While CsrA binding to Csr-family sRNAs plays a regulatory role in many *Gammaproteobacteria*, some organisms employ a protein that antagonizes CsrA activity. FliW was the first example of this kind of protein, which is present in the gram-positive firmicute *Bacillus subtilis* (Mukherjee et al., 2011). *B. subtilis* CsrA inhibits translation of *hag* mRNA that encodes the flagellar filament protein. Free FliW directly inhibits CsrA from binding to and occluding the SD sequence of the *hag* transcript and, thus, releaves CsrA-mediated repression of *hag* translation (Yakhnin et al., 2007; Mukherjee et al., 2011). The Hag flagellin, together with FliW and CsrA, are involved in a partner-switching mechanism promoting Hag homeostasis (Mukherjee et al., 2011; Figure 4). Unlike CsrB/C sRNAs, FliW does not interact with the RNA-binding amino acid residues in the RNA-binding pocket of CsrA. Moreover, structural analysis of FliW-CsrA complex revealed that FliW interacts with the extended C-terminus of CsrA (Altegoer et al., 2016; Mukherjee et al., 2016). As such, FliW has been proposed to allosterically antagonize CsrA *via* a noncompetitive mechanism (Altegoer et al., 2016; Mukherjee et al., 2016). A recent study revealed that the Hag-FliW-CsrA^dimer^ system functions at a nearly equimolar ratio. The components participate in a three-node negative-feedback loop that maintains Hag homeostasis in the cytoplasm, with similarities to toxin-antitoxin systems (Oshiro et al., 2019). Interestingly, FliW appears to be widely dispersed among motile bacteria and has been found to interact with CsrA in the gram-negative bacterium *Campylobacterium jejuni* (Dugar et al., 2016; Radomska et al., 2016; Li et al., 2018). However, *fliW* is notably absent from *Gammaproteobacteria*, and the presence of this gene is anti-correlated with genes encoding BarA-UvrY and their homologs, which are required for transcription of Csr-family sRNAs in many species (Zere et al., 2015). Whether FliW and sRNAs function together to modulate CsrA activity in any bacterial species remains to be determined.

In enteropathogenic *E. coli* (EPEC), CsrA regulates genes of the locus of enterocyte effacement, the Lee locus, which are involved in the Type III secretion system (T3SS) of this bacterium (Bhatt et al., 2009). Furthermore, CsrA binds to CesT in this bacterium, a chaperone protein needed for colonization of host intestinal epithelial cells *via* the T3SS (Katsowich et al., 2017). Although FliW and CesT are both protein antagonists of CsrA, they employ distinct mechanisms for performing this role. The CesT binding region in CsrA extensively overlaps the RNA-binding pockets, which is indicative of a competitive antagonism, similar to that of the Csr sRNAs (Ye et al., 2018). Furthermore, the CsrA-CesT interaction was apparent only at high local concentrations of CesT, suggesting that it enables bacteria to redirect gene expression after the effector proteins with which CesT interacts, especially Tir, have been injected into the host (Ye et al., 2018; Elbaz et al., 2019). Because the regulatory interactions of Tir-CesT-CsrA are responsible for the remodeling of virulence and metabolic gene expression, this regulation is likely required for EPEC survival at the host intestinal epithelium.

## Complex Feedback Loops and Circuitry of the Csr System

As described above, CsrA activity in *E. coli* is regulated by sequestration by its sRNA antagonists, CsrB and CsrC. The short half-lives of these RNAs (Gudapaty et al., 2001; Weilbacher et al., 2003; Leng et al., 2016) allow for rapid adjustments of CsrA activity in response to factors affecting sRNA transcription and stability. Antagonism of the stable CsrA protein by these RNAs occurs even during growth arrest, making this regulation robust to variations in growth rate (Adamson and Lim, 2013). Additionally, the presence of two negative regulators of CsrA activity implies a functional redundancy, which is known to decrease nongenetic noise and increase regulatory precision (Kafri et al., 2006). In both *E. coli* and *S. enterica* serovar Typhimurium, transcription of *csrB* (but not *csrC*) is activated by integration host factor (IHF), suggesting that the sRNAs may also have distinct regulatory roles in some species (Martínez et al., 2014; Zere et al., 2015).

**Figure 5:**
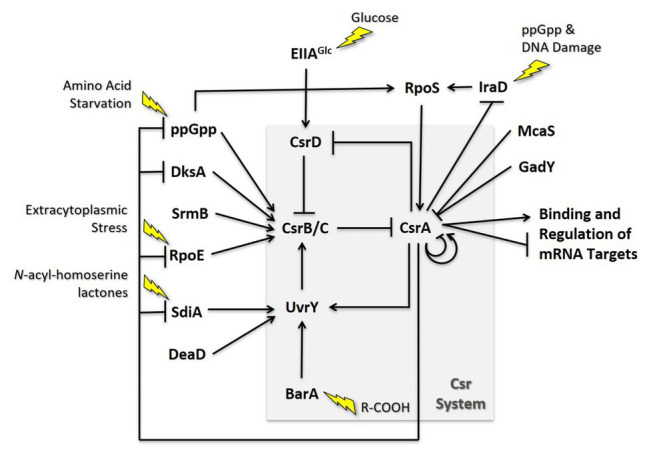
Regulatory circuitry impacting the Csr system of *E. coli*. These interactions are discussed in the text.

The *Escherichia coli* Csr system is comprised of multiple positive and negative feedback loops that tightly control the levels of each molecular component (Figure 5). For example, CsrA indirectly activates transcription of its sRNA antagonists through its effects on the BarA-UvrY TCS (Gudapaty et al., 2001; Suzuki et al., 2002; Weilbacher et al., 2003). Phosphorylated UvrY activates expression of CsrB/C, and CsrA has positive effects on *uvrY* expression at both the transcriptional and translational levels (Camacho et al., 2015; Zere et al., 2015). CsrA is also necessary for proper switching of the membrane-bound BarA protein from its phosphatase to kinase activity (Camacho et al., 2015). Because CsrB and CsrC depend on free CsrA for their synthesis while also antagonizing its activity, disruption of the expression of either regulatory RNA results in a compensatory increase in the level of the other (Weilbacher et al., 2003; Suzuki et al., 2006). Additionally, CsrA in both *E. coli* and *Salmonella* indirectly stabilizes CsrB/C by repressing the expression of CsrD, which targets these antagonists for RNase E-dependent degradation (Suzuki et al., 2006; Jonas et al., 2008). These negative feedback loops support rapid signal propagation and demonstrably reduce the time required for the system to achieve a steady state (Adamson and Lim, 2013).

CsrA also directly and indirectly regulates its own expression. In *Escherichia coli*, *csrA* expression is complex, involving five promoters, two sigma factors, and four CsrA binding sites. Transcription from σ^s^-dependent promoter 3 is indirectly activated by CsrA and is largely responsible for the substantial increase in *csrA* expression when cells transition to stationary-phase growth. Furthermore, CsrA binds to four sites in its own leader, one of which overlaps the SD sequence, and thereby inhibits its own translation (Yakhnin et al., 2011b). This incoherent feedforward loop likely enhances CsrA expression in response to σ^s^-inducing stressors while providing another mechanism for rapid repression when free CsrA levels are high. The structure of the Csr system and its interrelated regulatory pathways suggest a multifaceted arrangement of autoregulation, a common characteristic of systems that maintain homeostasis. Its complex regulatory circuitry involves multiple network motifs that tightly control the levels and/or activity of each molecular component. This arrangement likely reduces cell-to-cell variability under a given growth condition, supports rapid intracellular signaling, and may limit stochastic fluctuations in a system responsible for both responding to and coordinating the influence other signaling networks (Alon, 2007; Adamson and Lim, 2013; Potts et al., 2017).

## Interactions of the Csr System with Other Regulatory Systems

Among the most striking findings to come to light from transcriptomics and other systems-based studies are that CsrA regulates the expression of dozens of transcription factors and other regulatory genes (Edwards et al., 2011; Potts et al., 2017, 2018, 2019; Sowa et al., 2017). These effects expand the regulatory influence of CsrA *via* indirect effects on the structural genes that respond to the CsrA-controlled regulators. These findings illustrate the importance of CsrA, and posttranscriptional regulation in general, in determining the complex structure of bacterial regulatory networks. Although the biology of many of these regulatory interactions has not been elucidated, a few examples of how the Csr system interacts with other regulatory factors and systems have been studied. These include direct roles of CsrA in controlling expression of *hfq*, *pnp*, *sdiA*, and *nhaR* (Baker et al., 2007; Yakhnin et al., 2011a; Pannuri et al., 2012; Park et al., 2015), as well as examples in which Csr system components have been shown to both control and be controlled by other regulators.

### Multitier Regulation of Gene Expression

**Figure 6:**
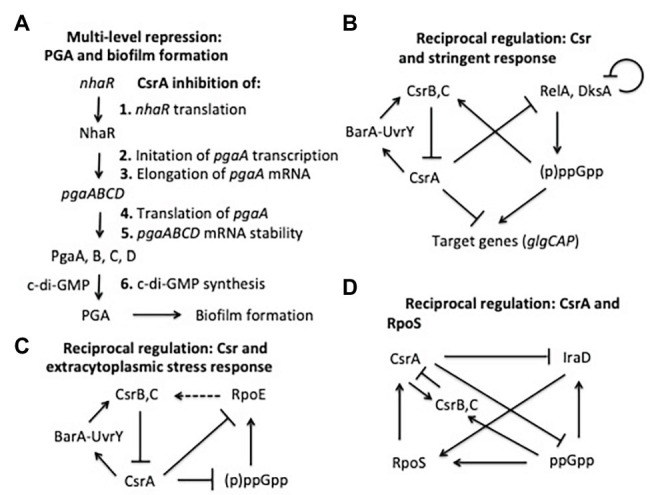
Regulatory circuits involving CsrA interactions with other regulators. The models shown here are described in the text.

CsrA was one of the first regulators of bacterial biofilm formation to be identified (Romeo et al., 1993; Jackson et al., 2002). Since that time, it has been found to play a complex regulatory role in biofilm formation, accomplished by interactions at several levels (Figure 6A). The major influence of CsrA is to act as a posttranscriptional repressor of the *pgaABCD* operon, which is needed for the biosynthesis and secretion of an adhesive glycosaminoglycan, variously referred to as PGA, PNAG, dPNAG, or PIA (Wang et al., 2004, 2005, 2016). As described above, binding to *pgaA* leader mRNA leads to repression of its translation, destabilization, and transcription termination. Besides interacting directly with *pgaABCD* mRNA, CsrA represses the translation of a LysR-family transcription factor, NhaR, which binds upstream of the single *pgaABCD* promoter and activates transcription in response to high pH and [Na^+^] (Goller et al., 2006; Pannuri et al., 2012). Finally, CsrA represses the expression of genes needed for production of c-di-GMP, an alarmone that allosterically activates the polymerization of PGA by the PgaCD protein and, thus, enhances biofilm formation (Jonas et al., 2008; Steiner et al., 2013). These findings demonstrate that CsrA effects multi-tier regulation of PGA biosynthesis and biofilm circuitry (Romeo et al., 2013; Figure 6A).

As previously discussed, the regulation of *flhDC* expression in *E. coli* represented the first mechanism of genetic activation by CsrA to be elucidated. The *flhDC* operon encodes the two subunits of a transcription factor, FlhD_4_C_2_ that initiates the three-tier cascade of gene expression needed for temporal development of the flagellum and chemotaxis in *E. coli* and its relatives (Lee et al., 2011; Fitzgerald et al., 2014). A *csrA::kan* transposon mutation was found to eliminate flagellum production and motility by reducing the expression of *flhDC* below the threshold necessary for acquiring motility (Wei et al., 2001). Importantly, this defect could be corrected by ectopic expression of *csrA*, as well as *flhDC*, suggesting that the main role of CsrA in motility is to activate *flhDC* expression. Nevertheless, transcriptomics studies revealed that *flhDC* mRNA is not the only target of CsrA that is involved in *E. coli* motility; CsrA activates the expression of multiple motility genes (Potts et al., 2017). FlhD_4_C_2_ itself directly activates transcription of second tier genes of the motility cascade of gene expression, including *fliA*, which encodes σ^28^. This alternative sigma factor is required for transcription of the third tier of motility genes. The direct effects of CsrA on FlhD_4_C_2_ may impact the expression of all motility genes. However, CLIP-seq analyses and CsrA:RNA co-purification followed by RNA-seq also identified transcripts of a variety of flagellar genes in addition to *flhDC* as probable direct targets of CsrA binding (Edwards et al., 2011; Potts et al., 2017). These include the regulatory genes *fliA* (σ^28^) and *flgK*, a morphogene involved in controlling the switch from secretion of hook proteins to filament proteins during flagellum assembly. Together, these findings indicate that CsrA plays a complex role as a multi-tier regulator within the motility cascade, with biological implications remaining to be determined.

### Reciprocal Interactions With Transcriptional Regulatory Systems

As mentioned above, the stringent response system, based on the nucleotide alarmone (p)ppGpp, stimulates transcription of CsrB and CsrC sRNAs. In addition, CsrA represses *relA* and *dksA* expression, and (p)ppGpp levels are elevated during the stringent response in a *csrA* mutant relative to the WT background (Edwards et al., 2011). These regulatory effects define reciprocal regulatory circuitry, through which the Csr system reinforces the effects of the stringent response system. Expression of CsrB/C is activated by ppGpp when cells are starved for amino acids or other nutrients, thus reducing CsrA activity, increasing *relA* expression and the potential for additional (p)ppGpp production (Jonas and Melefors, 2009; Edwards et al., 2011; Figure 6B). Similar to ppGpp, DksA acts by binding to RNA polymerase, and often potentiates the effects of ppGpp (Paul et al., 2004; Aberg et al., 2009). CsrA also posttranscriptionally represses expression of *dksA*, although the DksA protein feedback inhibits its own transcription, which can mask the effects of CsrA (Chandrangsu et al., 2011; Edwards et al., 2011).

Another example in which the Csr system interacts reciprocally with a stress response system is found in the extracytoplasmic stress response system (Figure 6C). The extracytoplasmic stress system detects misfolded proteins in the periplasm, relying on the sigma factor σ^E^ (RpoE) for transcription of stress response genes when this occurs (Ades, 2008; Yakhnin et al., 2017). In the absence of stress, σ^E^ is largely sequestered at the cytoplasmic membrane by its interaction with the membrane-bound anti-sigma factor RseA. Upon stress, proteolytic cleavage of RseA leads to release of σ^E^, making it available for interaction with the RNA polymerase core enzyme. RNA-seq studies first suggested that CsrA binds to the *rpoE* transcript (Edwards et al., 2011). Biochemical studies revealed that CsrA binds to three sites in *rpoE* mRNA, two of which overlap the SD sequence and the initiation codon, leading to repression of translation (Yakhnin et al., 2017). In addition, RpoE indirectly activates transcription of CsrB and CsrC sRNAs, although the mechanisms responsible for this remain to be determined. This circuitry was seen as increasing CsrB/C synthesis during extracytoplasmic stress, thus limiting CsrA activity and leading to an increase in *rpoE* translation. When the stress has been resolved, CsrB/C levels should decline and CsrA activity should increase, helping to reestablish σ^E^ levels to a pre-stress state. Because (p)ppGpp and DksA activate expression of *rpoE* independently of RseA under nutrient limitation (Costanzo and Ades, 2006; Costanzo et al., 2008), this pathway *via* σ^E^ may also contribute to the increase in CsrB/C sRNA levels during the stringent response (Edwards et al., 2011; Zere et al., 2015). Restoration of nutrients should decrease (p)ppGpp levels, helping to reset the system.

Among the most global of the *E. coli* regulatory factors is the alternative sigma factor σ^s^ (RpoS), which activates gene expression upon entry into the stationary phase of growth and exposure to a variety of physicochemical stresses (Battesti et al., 2011). Regulation of σ^s^ levels is complex and multifactorial, including inhibition of its turnover by anti-adapter proteins that prevent the RssB adapter protein from triggering its cleavage by ClpXP protease under a variety of stresses. Expression of the anti-adapter protein IraD is activated by ppGpp accumulation and DNA damage (Merrikh et al., 2009a,b). CsrA was found to interact *in vivo* with the *iraD* transcript by using CLIP-seq analysis and was seen to inhibit its expression (Potts et al., 2017). This regulation involved a new mechanism, in which CsrA directly repressed the translation of a short open reading frame, ORF27 (*idlP*), located immediately upstream and slightly overlapping the *iraD* coding region (Park et al., 2017). Repression of *iraD* translation by CsrA was mediated entirely through translational coupling of *iraD* to *idlP*. RpoS acts at two of the five *csrA* promoters, activating CsrA transcription upon entry into stationary phase (Yakhnin et al., 2011b). CsrB and CsrC sRNAs also accumulate at this time, each of which can sequester multiple CsrA proteins (Gudapaty et al., 2001; Weilbacher et al., 2003). The latter effect seemingly dominates, such that overall CsrA activity decreases while CsrA protein accumulates. Thus, RpoS should be stabilized in the stationary phase *via* effects of the Csr system on IraD. Similarly, under stress-related induction of ppGpp production, CsrA expression should be activated *via* RpoS. Coordinately, CsrA activity will be decreased by the ppGpp-dependent increase in the accumulation of CsrB/C (Edwards et al., 2011), which should override the increase in CsrA protein. Activation of CsrA transcription *via* RpoS as cultures enter stationary phase would seem to ensure that sufficient CsrA will be available to rapidly restore gene expression needed for growth resumption upon replenishment of nutrients or relief of stress, although this remains to be demonstrated experimentally (Figure 6D).

A final example of reciprocal regulatory interactions of Csr with other regulatory systems involves the *E. coli N*-acyl-homoserine lactone (HSL) receptor SdiA (Dyszel et al., 2010). As described above, CsrA represses translation of *sdiA*, while genetic studies indicate that SdiA activates transcription of the gene encoding UvrY, the key transcriptional activator of *csrB/C* (Suzuki et al., 2002). The resulting feedback activation loop is consistent with autoinduction, a well-described feature of HSLs that mediate quorum sensing. However, SdiA is an orphan HSL receptor in *E. coli* and closely related species, which cannot synthesize HSLs. While SdiA maintains weak activity in the absence of HSL, binding to cognate HSLs of other species improves its function and permits sensing of the microbial environment (Smith and Ahmer, 2003; Dyszel et al., 2010; Venturi and Ahmer, 2015). The finding that HSLs produced by other species act as a signal to *E. coli via* SdiA, with important effects on physiology (Hughes et al., 2010; Sperandio, 2010; Lu et al., 2017), suggests that the Csr system may participate in the regulation of bacterial interspecies communications, in addition to its well-studied roles in virulence and host-microbe interactions (Vakulskas et al., 2015).

## New Connections From Systems-Based Approaches

Systems-based methodologies provide powerful approaches for investigating the roles of global regulatory factors, such as CsrA. Integrated systems approaches, which combine two or more complementary techniques applied to isogenic strains, grown under uniform conditions, have proven especially useful for generating hypotheses to guide the discovery of new regulatory roles, mechanisms, and circuitry. Integrated transcriptomics allowed global regulatory effects of CsrA on transcript levels, translation, and/or RNA stability to be analyzed in the context of the RNA binding interactions that may underlie the effects. While most of the vast information gathered in such studies needs to be confirmed and extended before the causal mechanisms and circuitry are understood, the implications of such findings for the regulatory landscape of CsrA are profound.

A study using RNA-seq to identify cellular RNAs that copurify with CsrA, coupled with proteomics analyses to determine the global effects of a *csrA* mutation on protein levels provided the impetus for investigating the interconnecting roles of the Csr and stringent response systems, described above (Edwards et al., 2011). A more recent integrated transcriptomics study combined CLIP-Seq for monitoring *in vivo* RNA binding interactions of CsrA with ribosome profiling to quantitatively monitor the positioning of translating ribosomes and RNA-Seq to reveal both steady state transcript levels and transcript stability in WT and *csrA* mutant strains (Potts et al., 2017). This study uncovered many new potential roles of CsrA, e.g., in regulating iron metabolism, toxin-antitoxin systems, membrane homeostasis, and expression of many regulatory factors such as global transcription factors and sRNAs. Evidence that CsrA binds *in vivo* to mRNA encoding *iraD* and affects the level of this transcript compelled the *iraD* studies described above (Park et al., 2017). CsrA affected the expression of several genes involved in iron homeostasis (Potts et al., 2017). A follow-up study used *in vivo* and *in vitro* approaches to demonstrate that CsrA binding interactions directly repress the translation of genes for three iron storage proteins, *ftnB*, *bfr*, and *dps*, which are expressed in the stationary phase of growth and affect oxidative stress. In addition, repression of *ftnB* and *bfr* by CsrA was found to be essential for normal cell growth under iron-limiting conditions (Pourciau et al., 2019).

The integrated transcriptomics study also provided evidence that CsrA regulates diverse new targets including transcriptional regulators, RNases, and sRNAs (Potts et al., 2017). Transcriptional factors identified (e.g., *fliA*, *lrp*, *cra*, and *soxS*) regulate distinct physiological properties ranging from motility, amino acid metabolism, and central carbon metabolism, to resistance to a variety of stresses. Lrp alone regulates at least 10% of *E. coli*’s genome (Tani et al., 2002), suggestive of a major new route of global regulation by CsrA. The finding that CsrA regulated genes for ribonucleases (including *rng*, *rnb*, and *orn)*, which along with its known role in regulating *pnp* (Park et al., 2015), may help to explain the contrasting negative effects of CsrA on overall RNA translation and abundance vs. its action as a stabilizer of total RNA decay (Esquerré et al., 2016; Potts et al., 2017, 2018). Of the new sRNA interactions identified, CsrA bound with high affinity to GadY, Spot42, GcvB, and MicL (Potts et al., 2017). These findings call for research into the physiological and mechanistic implications and the potential CsrA-dependent regulatory circuits that they create.

Another recent integrated transcriptomics approach demonstrated the flexibility of CsrA as a condition-specific regulator in *Salmonella*. The study employed ribosome profiling and RNA-seq to examine the effects of CsrA on ribosome occupancy, transcript levels, and transcript stability in the rich medium Luria Broth (LB) and the acidic and nutrient limiting medium mLPM (Potts et al., 2019). These media favor the expression of genes in *Salmonella* pathogenicity islands SPI-1 and SPI-2, respectively, which are primarily associated with growth in the intestine vs. the *Salmonella*-containing vacuole of the macrophage. While certain genes were regulated under both growth conditions, others were subject to condition-specific regulation by CsrA, including key regulators involved in stress and virulence such as *rpoS*, *slyA*, and *spvR*. The results from this study illustrated how the Csr system may act as a physiological switch in responding to external stimuli, with potential regulatory consequences for virulence, metabolism, and stress responses.

CLIP-seq studies from both *E. coli* and *Salmonella* revealed the unexpected finding that CsrA binds not only to mRNA leader regions but also binds extensively within the coding regions of mRNAs (Holmqvist et al., 2016; Potts et al., 2017). Furthermore, ribosome pause sites were significantly enriched near CsrA crosslinking sites (Potts et al., 2017). Because the ribosome profiling pattern was unaltered in a *csrA* mutant strain, CsrA does not grossly affect ribosome pausing. However, ribosome pausing may facilitate CsrA binding to sites that would otherwise be protected by active translation. Whether CsrA binding near ribosome pause sites has regulatory consequences is of considerable interest, as ribosome pausing occurs during nutrient limitation and other stresses (Subramaniam et al., 2014), and might provide a means of altering the availability of free CsrA protein under such conditions.

An approach combining RNA-seq with epistasis analysis (Epi-seq) was used to distinguish two alternative models for how the CsrD protein exerts global effects on gene expression: by altering the stability of CsrB/C RNAs and, thus, CsrA activity (described above), or by acting as a transcription factor that regulates numerous transcripts, as proposed based on observations from a transcriptomics study (Esquerré et al., 2016). Because global effects of CsrA on transcripts remained mostly unaltered by deletion of *csrD*, while disruption of *csrA* eliminated all effects of *csrD* and deletion of *csrB* and *csrC* eliminated almost all effects, it was concluded that the CsrD acts as a global regulator through its role in CsrB/C sRNA decay.

Metabolomics studies of CsrA have revealed striking changes in metabolite levels upon *csrA* disruption, some of which were predictable from earlier investigations, but many of which were unanticipated. Studies in *E. coli* K-12 showed that metabolites in glycolysis upstream of phosphofructokinase (PfkA) accumulate (Morin et al., 2016), in agreement with CsrA effects on this enzyme and its transcript (Sabnis et al., 1995; Potts et al., 2017). Studies in *E. coli* Nissle 1917 revealed that the influence of CsrA on metabolite levels and metabolic flux were conditional, differing dependent upon the metabolic fate of the carbon source that was used for growth (Revelles et al., 2013). A study conducted in EPEC showed that deletion of *csrA* resulted in a significant change in over half of the metabolites observed (Berndt et al., 2019). In line with previous work conducted in CsrA transposon insertion mutants, the deletion led to an accumulation of glycogen and fructose-6-phosphate (Berndt et al., 2019).

An unanticipated finding from the EPEC metabolomics study was that nucleotides were depleted, while nucleosides and nucleobases accumulated in the *csrA* mutant. These changes were associated with a corresponding drop in expression of nucleotide synthesis genes such as *cdd*, *rihB*, and *deoA* (Berndt et al., 2019). These findings might help to explain why *csrA* deletion mutants often have severe growth defects or are unable to grow (Altier et al., 2000; Timmermans and Van Melderen, 2009; Mey et al., 2015). Furthermore, while aromatic amino acids were depleted, an intermediate in their synthesis, shikimate was observed to have accumulated drastically and the expression of shikimate kinase (AroL) dropped significantly. Finally, colanic acid accumulation was accompanied by a large increase in the expression of genes in the colanic biosynthesis pathway in the *csrA* mutant (Berndt et al., 2019). The precise mechanisms for these metabolic alterations are not fully understood.

## Conclusions and Perspectives

Posttranscriptional regulation by the RNA-binding protein CsrA in *E. coli* and related bacterial Csr systems is crucial for maintenance of robust growth and for orchestrating major lifestyle decisions in response to nutritional and stress conditions. Binding interactions of CsrA-family proteins with RNA targets are known in atomic detail and give rise to diverse regulatory mechanisms. Interactions of CsrA with Csr-family sRNAs have long been known to sequester this protein and, thus, modulate its activity and the mechanisms and physiology behind Csr sRNA synthesis and turnover are partially understood. In contrast, CsrA interactions with multiple basepairing sRNAs, recently identified by transcriptomics analyses, are poorly understood, requiring investigation of both mechanisms and biological functions. The recent discovery of extensive binding of CsrA deep within coding regions of mRNAs in proximity to ribosome pause sites also requires further examination. CsrA regulates dozens of regulators and the Csr system has been found to participate in complex circuitry with some of these regulators. These interactions extend the regulatory reach of the Csr system and allow the CsrA regulon to respond to broader aspects of physiology and metabolism. While Csr systems are among the best-known bacterial posttranscriptional regulators, many new discoveries will be required for a full appreciation of their pervasive roles.

## Author Contributions

TR conceived and outlined this review. All authors contributed to the article and approved the submitted version.

### Conflict of Interest

The authors declare that this review was written in the absence of any commercial or financial relationships that could be construed as a potential conflict of interest.
